# Evaluation of BMMSCs-EPCs sheets for repairing alveolar bone defects in ovariectomized rats

**DOI:** 10.1038/s41598-017-16404-3

**Published:** 2017-11-29

**Authors:** Yi Wen, Hongxu Yang, Yanli Liu, Qian Liu, Axian Wang, Yin Ding, Zuolin Jin

**Affiliations:** 1 State Key Laboratory of Military Stomatology, National Clinical Research Center for Oral Diseases, Shaanxi Clinical Research Center for Oral Diseases, Department of Orthodontics, School of Stomatology, the Fourth Military Medical University, 145 Changle West Road, Xi’an, 710032 China; 2 State Key Laboratory of Military Stomatology, National Clinical Research Center for Oral Diseases, Shaanxi International Joint Research Center for Oral Diseases, Department of Oral Anatomy and Physiology and TMD, School of Stomatology, the Fourth Military Medical University, 145 Changle West Road, Xi’an, 710032 China; 3 State Key Laboratory of Military Stomatology, National Clinical Research Center for Oral Diseases, Shaanxi International Joint Research Center for Oral Diseases, Department of General Dentistry and Emergency, School of Stomatology, the Fourth Military Medical University, 145 Changle West Road, Xi’an, 710032 China

## Abstract

The aim of this paper is to investigate the effect that bone marrow mesenchymal stem cells (BMMSCs) - endothelial progenitor cells (EPCs), BMMSCs and EPCs sheets have on repairing maxillary alveolar defects in ovariectomized (OVX) rats. In this study, after identification using multi-lineage differentiation and flow cytometry, BMMSCs and EPCs were isolated from female rats. The BMMSCs-EPCs, BMMSCs and EPCs sheets were detected by hematoxylin-eosin (H&E) staining, alkaline phosphatase (ALP) staining and qRT-PCR. Defects were created in maxillary alveoli and repaired with BMMSCs-EPCs, BMMSCs and EPCs sheets in OVX rats. The repair effects were determined by histological staining and micro-CT analysis at 2, 4 and 8 weeks after implantation. We aim to clarify whether BMMSCs-EPCs sheets are more effective in repairing alveolar bone defects than are BMMSCs and EPCs sheets in OVX rats. The results show that the osteogenic potential and the effect of bone repair are greater in the BMMSCs-EPCs sheet group and that this group has a higher ability to repair alveolar bone defects in OVX rats. These results suggest that BMMSCs-EPCs sheets have potential in clinical applications for treating humans with osteoporosis.

## Introduction

Postmenopausal osteoporosis (PMO) often occurs within 10 to 15 years after menopause, and it frequently results in alveolar bone resorption, periodontal tissue degeneration and the increased difficulty of tissue regeneration^1^. The absorption and osteogenesis of alveolar bone are balanced, and these two processes promote bone regeneration^2,3^. Significant bone mass loss will cause osteoporosis, leading to reduced bone mineral density and bone microstructure degradation. The higher incidence of osteoporosis in postmenopausal women often results in alveolar bone resorption and increased repair difficulty^1^. But there was few research for the alveolar bone resorption and defect in osteoporosis.

Alveolar bone defects have aroused widespread concern worldwide. Auto- and allo-transplantation are two main approaches to repair bone defects, but these approaches are limited by some factors, such as postoperative complications, the limited amounts of donor bone and the bone resorption of autografts^4,5^. Currently, there are several methods for bone repair, such as the application of bone transplantation, artificial bone and bone tissue engineering. Bone transplantation is the preferred method for repairing bone defect, especially for small defects^4^. Autologous bone grafts have several problems, such as increased surgical trauma and operation time, limited availability of bone, and difficult in meeting the requirements during operation^5^. Although the source of allograft bone is much more abundant, it may increase the risk of spreading the disease and the possibility of immune rejection. Artificial bone can satisfy the demand of graft bone mass and is a reasonable alternative, but it has some limitations, such as low mechanical strength, weak synosteosis, and poor biodegradability^5^. In contrast, bone tissue engineering is considered a viable alternative strategy that can overcome the shortcomings described above^6^. Bone marrow mesenchymal stem cells (BMMSCs) are the seed cells of bone tissue engineering, and the proliferation and osteogenesis abilities of BMMSCs are typically diminished in humans with osteoporosis^7^. Endothelial progenitor cells (EPCs) are the precursor cells of vascular endothelial cells (ECs), and they can develop and differentiate into peripheral blood cells and blood vessels^8^. However, the effect of a combination strategy with BMMSCs and EPCs in the repair of bone defect is unknown.

Bone healing and reconstruction depend on the generation of local capillaries, especially when the defect is large or the ability to regenerate tissue is poor. It is of great significance to improve bone tissue regeneration and repair by promoting angiogenesis. Angiogenesis is a key step in treating bone defects and ensures the supply of adequate nutrition, metabolite transfer and circulating progenitor cells. If the blood supply is not adequate at the beginning of the bone tissue transplantation, then the process of bone healing may be interrupted^8–10^. EPCs are the precursor cells of vascular ECs, and they can develop and differentiate into peripheral blood cells and blood vessels. In addition, EPCs exhibit homing ability, as well as proliferation, differentiation, and the formation of new blood vessels^11–14^.

Several studies have confirmed that estrogen can regulate the proliferation and migration of EPCs^15^. Mobilizing endogenous EPCs from bone marrow may be a feasible method that can not only increase the generation of new blood vessels but also be used to treat ischemic cardiovascular disease. Pro-inflammatory cytokines, growth factors, cytokines, and hormones can mobilize EPCs^16–18^, and EPCs can improve the function and activity of BMMSCs *in vitro*. BMMSCs have good proliferation and differentiation capacity under the appropriate experimental conditions *in vitro*. Additionally, the proliferation and differentiation of BMMSCs into osteoblast are regulated by many factors, including cytokines, hormones and growth factors. It is widely believed that the relation between ECs and osteoblasts can regulate bone metabolism by regulating blood vessel formation and cell differentiation^19–21^. Studies have also focused on the influence of ECs on osteoblast differentiation^22,23^. Accordingly, co-culture with EPCs *in vitro* may reverse the stem cell properties of BMMSCs and improve their effect on repairing alveolar bone defects in OVX rats.

Studies have confirmed that the osteogenesis of BMMSCs is affected by a lack of estrogen to some extent, which can increase apoptosis and decrease osteogenic differentiation^24,25^. The osteogenesis of BMMSCs for tissue regeneration is usually decreased due to a deficiency of estrogen. Improving the local microenvironment will be beneficial to the differentiation of BMMSCs and will promote the bone-healing progress. In this study, an OVX rat model is used to simulate osteoporosis in postmenopausal women. Additionally, this experiment aims to investigate how to improve the proliferation and differentiation of BMMSCs and thereby improve the repair of periodontal tissue defects in OVX rats. The cell sheets technique, micro-CT and histological staining were used, and this study may lay a theoretical foundation for improving bone regeneration and developing a new strategy for the alveolar bone healing progress. Taking advantage of the multilineage differentiation potential of BMMSCs and the angiogenesis promotion of EPCs, we developed a novel strategy to engineer vascularized bone grafts with BMMSCs-EPCs sheets. We hypothesized that BMMSCs-EPCs sheets would promote osteogenesis and allow well-vascularized bone graft formation without the use of foreign materials.

## Results

### Examination of osteoporosis model

After three months of ovariectomy, the rats in the experimental and control groups were sacrificed. The bilateral tibias were collected, and micro-CT was used. The proximal tibial metaphysis was scanned starting at approximately 1 mm distal to the growth plate (Supplementary Fig. 1a). Indicators, including bone volume fraction (BV/TV), trabecular thickness (Tb.Th) and trabecular number (Tb.N), in the experimental group were significantly lower than those in the control group but higher in trabecular separation (Tb.Sp) (Supplement Fig. 1b–e). After ovariectomy for three months, SD female rats showed severe osteoporosis, so the osteoporosis model was verified.

### Characterization of BMMSCs and EPCs

**Figure 1:**
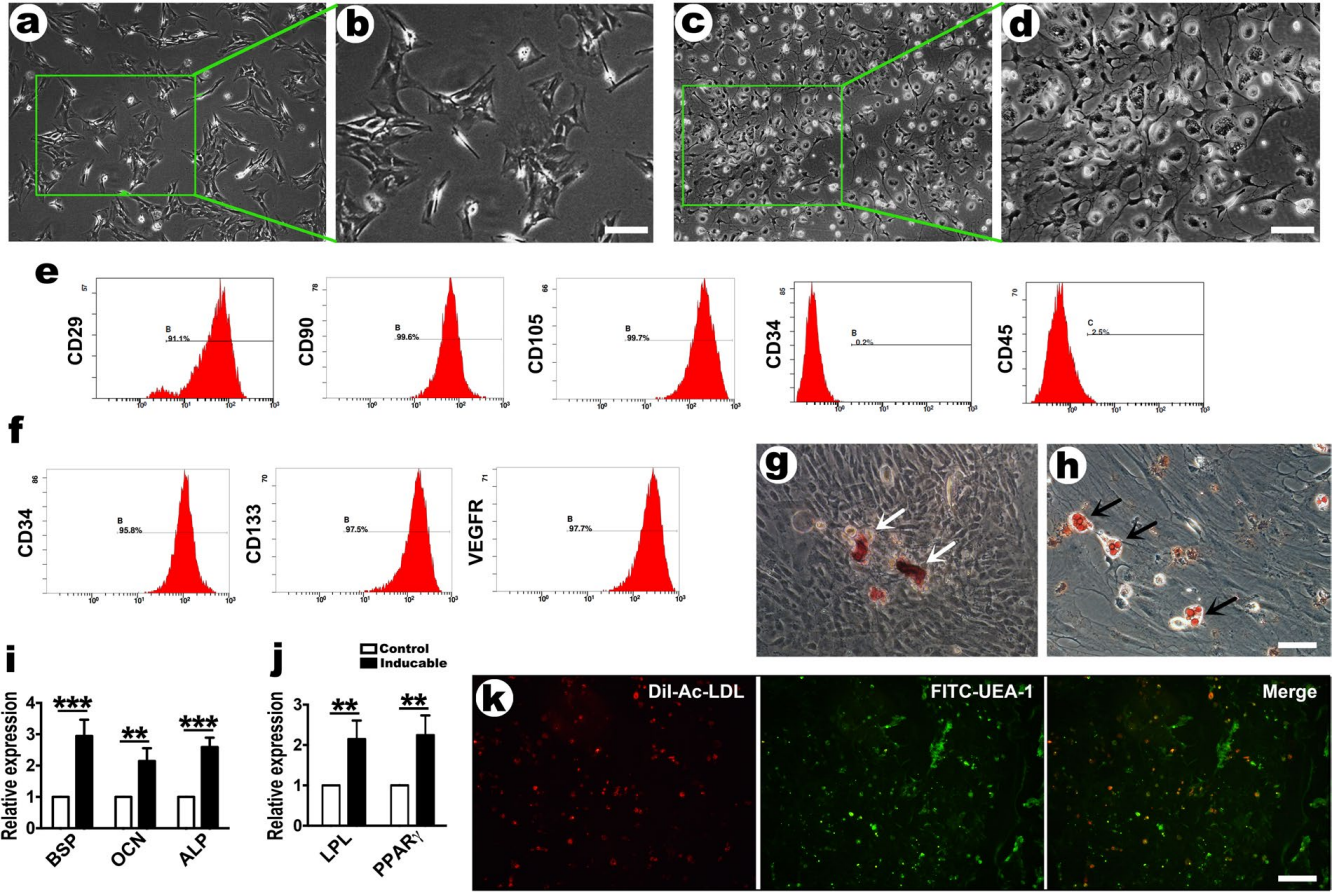
Cell culture and characterization of BMMSCs and EPCs. (**a**) The morphology of second passage BMMSCs (200×, bar represents 50 µm) and (**b**) shows higher magnification (400×, bar represents 25 µm). (**c**) The morphology of primary EPCs at 10 days for culture (200×, bar represents 50 µm) and (**d**) shows higher magnification (400×, bar represents 25 µm). (**e**) The expression of BMMSCs surface markers, CD29 (91.9%), CD90 (99.6%), CD105 (99.7%), CD34 (0.2%) and CD45 (2.5%), were detected by flow cytometry analysis. (**f**) The expression of EPCs surface markers, CD34 (95.8%), CD133 (97.5%), and VEGFR2 (97.7%), were detected by flow cytometry analysis. (**g**) Osteogenic differentiations of BMMSCs from passage two by alizarin red staining (400×, bar represents 25 µm). Three weeks after osteogenic induction, alizarin red staining revealed the formation of calcium nodules (white arrow). (**h**) Adipogenic differentiations of BMMSCs from passage two by oil red staining (400×, bar represents 25 µm). Two weeks after adipogenic induction, oil red O staining demonstrated intracellular lipid droplets (black arrow). (**i**) The mRNA expression levels of BSP, OCN, and ALP in the osteogenic groups. (**j**) The mRNA expression levels of LPL and PPARγ in the adipogenic group. (**k**) Cultured EPCs stained positive for both Dil-ac-LDL and FITC-UEA-I (400×, bar represents 25 µm). Data are presented as the means ± SD, n = 5. **p < 0.01 and ***p < 0.001 represent significant differences between the indicated columns.

After passage two, the BMMSCs exhibited irregular forms, with mostly polygonal or diamond shapes (Fig. 1a,b). Primary EPCs were cultured for 48 hours, and most of the cells were small and round. With the extension of the incubation time, the cells size increased with spindle morphology. After 9 to 10 days, some EPCs were rhombic (Fig. 1c,d). BMMSCs flow cytometry analysis implied that these cells were positive for the MSC markers CD-29, CD-90, and CD-105 and were negative for the hematopoietic stem cell markers CD-34 and CD-45 (Fig. 1e). Moreover, EPCs flow cytometry analysis showed that these cells expressed high levels of CD-34, CD-133 and VEGFR2 (Fig. 1f).

BMMSCs exhibited osteogenic and adipogenic differentiation. These cells were irregular or polygonal at the start of osteogenic induction. Within a few days, the cells showed an apparent response to the osteogenic induction medium with a pronounced morphological change. After two weeks, the accumulation of collagen increased, and the mineralized nodules increased over time. The nodules were obvious, and red calcium nodules (Fig. 1g) were observed under the microscope until approximately three weeks. At the beginning of adipogenic induction, the BMMSCs were apoptotic. After one week, round and small lipid droplets were observed in the cytoplasm. Subsequently, the lipid droplets in the cytoplasm were increasingly obvious and gradually converged, and the size of the BMMSCs expanded accordingly. After exposure to adipogenic induction for two weeks, different sizes of lipid droplets were observed in the cytoplasm under the microscope (Fig. 1h). Quantitative real-time PCR results indicated that under osteogenic or adipogenic culture conditions for 21 or 14 days, BMMSCs expressed higher levels of osteogenesis-related genes, such as *BSP*, *OCN* and *ALP* (Fig. 1i), and adipogenesis-related genes, such as *LPL* and *PPARγ* (Fig. 1j), than did cells grown in the control medium.

EPCs have the ability to simultaneously take up DiI-acetylated Low Density Lipoprotein (DiI-Ac-LDL) and bind Fluorescein Ulex Europaeus Agglutinin-1 (FITC-UEA-1). When EPCs took up DiI-ac-LDL, red fluorescence was observed under the fluorescence microscope. When EPCs bound FITC-UEA-1, green fluorescence was observed, and yellow fluorescence appeared after overlapping (Fig. 1k). The cells stained positively for both markers were considered differentiating EPCs.

### Direct co-culture of BMMSCs and EPCs

**Figure 2:**
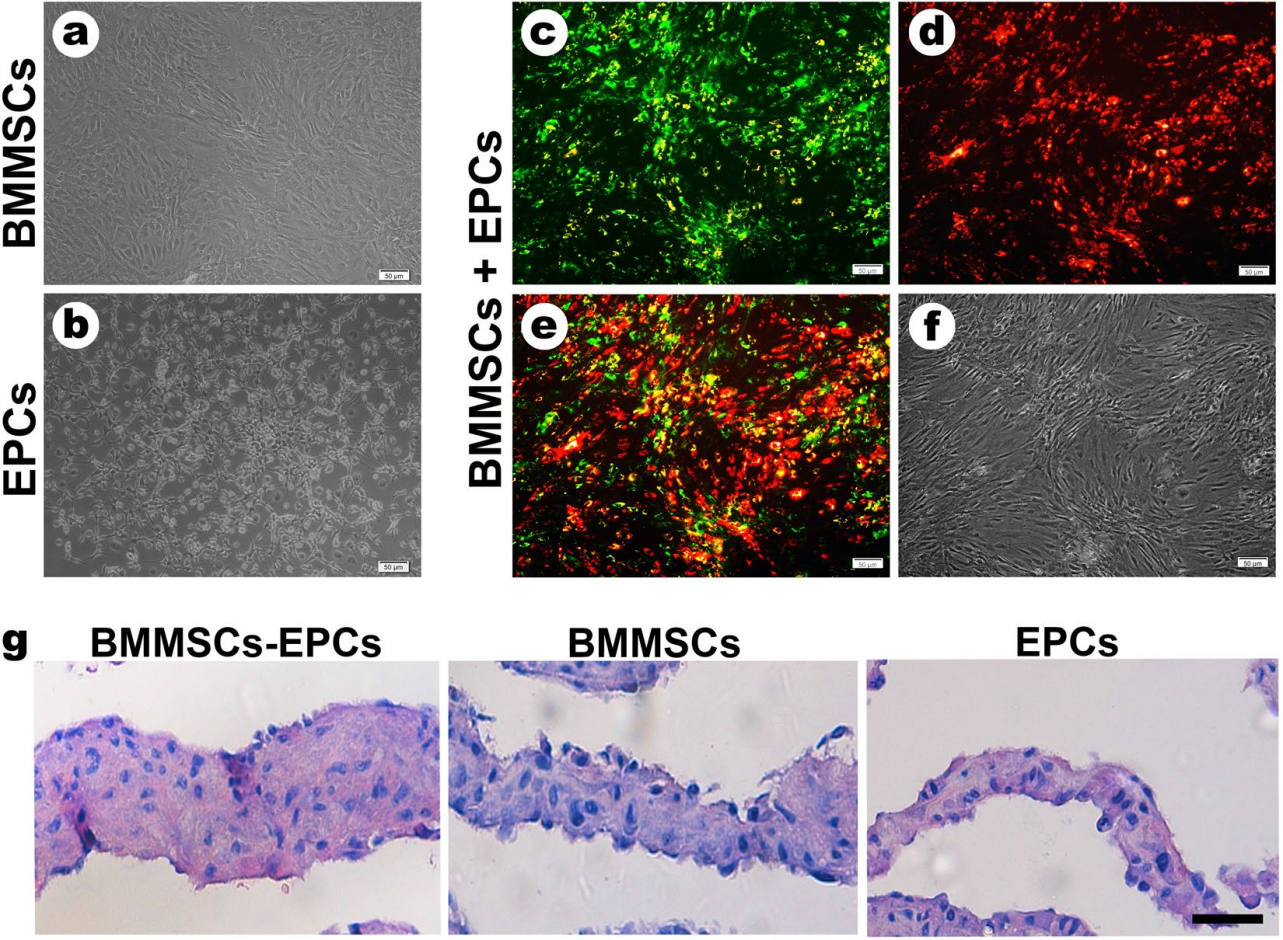
Cell morphology and observation of cell sheets. (**a**) The morphology of BMMSCs sheet under microscope. (**b**) The morphology of EPCs sheet under microscope. (**c**) BMMSCs-EPCs sheet were stained by DiO (green) for BMMSCs. (**d**) BMMSCs-EPCs sheet were stained by DiL (red) for EPCs. (**e**) Double fluorescent staining showed yellow fluorescent and clarified that two types of cells had contact during direct culture. (**f**) The morphology of BMMSCs-EPCs sheet under a microscope. (**g**) H&E staining showed the morphology and thickness of BMMSCs-EPCs sheets, BMMSCs sheets and EPCs sheets (400×, bar represents 25 µm).

Twenty-four hours after co-culture, BMMSCs and EPCs were attached to the flask (Fig. 2a,b). BMMSCs were stained with green fluorescence by the DiO cell marker (Fig. 2c), and EPCs were stained by the DiL (Fig. 2d) cell marker. Two types of cells were co-cultured, and they showed well growth, normal cell morphology and close arrangement. Additionally, it can be seen that the double fluorescent staining showed yellow fluorescence and signified two types of cells in contact during the direct cultivation under fluorescence microscopy (Fig. 2e,f).

### Properties of BMMSCs-EPCs, BMMSCs and EPCs sheet

**Figure 3:**
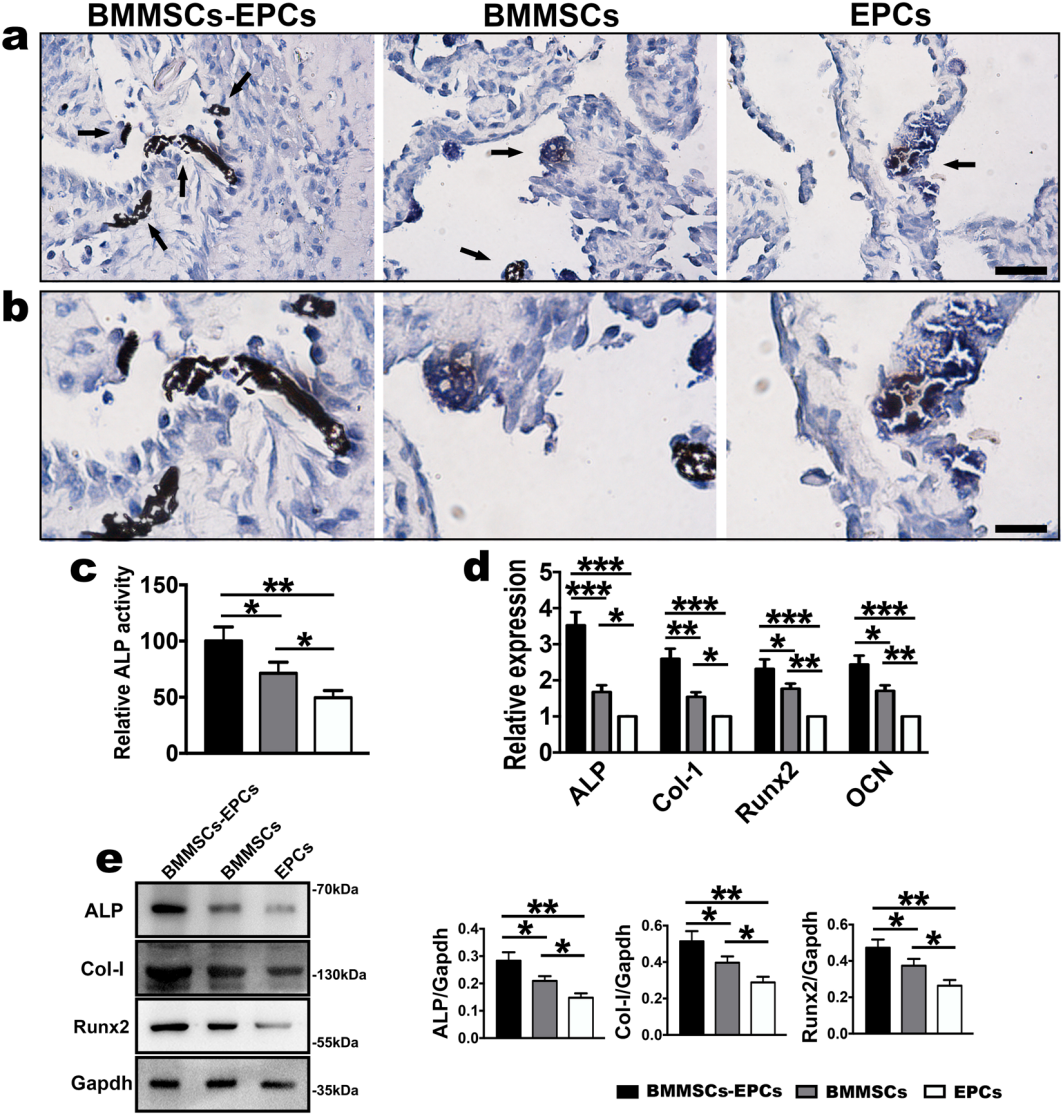
Characterization of BMMSCs-EPCs sheet, BMMSCs sheet, and EPCs sheet. (**a**) ALP staining was performed to show the calcium-rich granules (black arrow) on BMMSCs-EPCs sheet, BMMSCs sheet, and EPCs sheet (200×, bar represents 50 µm) and (**b**) shows the higher magnification (400×, bar represents 25 µm). (**c**) ALP activity assay of every cell sheets. (**d**) The osteogenesis-related mRNA expression of ALP, Col-1, Runx2 and OCN in BMMSCs-EPCs sheet, BMMSCs sheet and EPCs sheet. (**e**) Protein expression level of ALP, Col-1, and Runx2 by western blotting of BMMSCs-EPCs sheet, BMMSCs sheet and EPCs sheet. Data are presented as the means ± SD, n = 5. *p < 0.05 and **p < 0.01 represent significant differences between the indicated columns.

BMMSCs-EPCs, EPCs and BMMSCs sheets were fixed for H&E staining and ALP staining. H&E staining revealed that the cell sheets consisted of several cells layers surrounded by an abundant extracellular matrix (ECM). In addition, the cells were arranged in order. BMMSCs-EPCs sheets were composed of more layers of cells than were BMMSCs and EPCs sheets (Fig. 2g). ALP staining and activity assayed demonstrated that the three groups of cell sheets exhibited osteogenesis (Fig. 3a–c). Compared with BMMSCs and EPCs sheets, BMMSCs-EPCs sheets had a larger area of mineralized nodules, indicating greater osteogenic differentiation (Fig. 3b). Quantitative real-time PCR revealed that the expression levels of the osteogenic-associated genes ALP, Col-1, Runx2 and OCN were significantly higher in BMMSCs-EPCs sheets than in BMMSCs and EPCs sheets (Fig. 3d). Moreover, the expression levels of these genes were higher in BMMSCs sheets than they were in EPCs sheets. These expression changes were confirmed by the protein western blot data, which indicated that the levels of ALP, Col-1 and Runx2 proteins were increased in BMMSCs-EPCs sheets (Fig. 3e).

### Repairing effect of the alveolar bone defect in OVX rats

**Figure 4:**
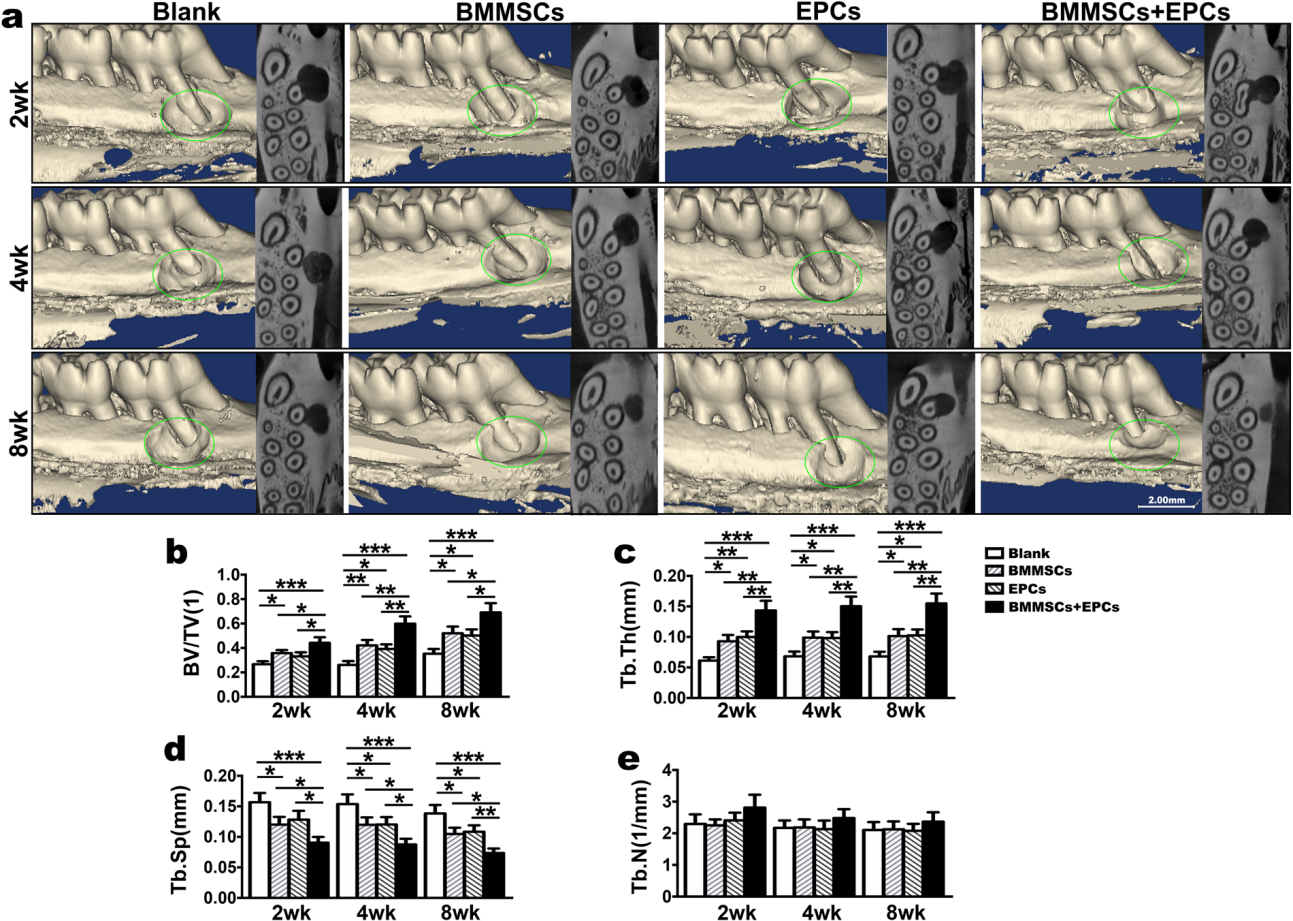
Micro-CT analysis of bone defects of OVX rats. (**a**) Repair effect of bone defects of OVX rats treated with BMMSCs-EPCs sheet, BMMSCs sheets, EPCs sheets and blank control at 2, 4 and 8 weeks after transplantation representation in 3D image and horizontal cross section. The green circle displays the size of bone defect model. (**b**) BV/TV, (**c**) Tb.Th, (**d**) Tb.N and (**e**) Tb. Sp analysis of bone defects of OVX rats treated with BMMSCs-EPCs sheet, BMMSCs sheets, EPCs sheets and blank control group with each other. Data are presented as the means ± SD, n = 5. *p < 0.05, **p < 0.01 and ***p < 0.001 represent significant differences between the indicated columns.

**Figure 5:**
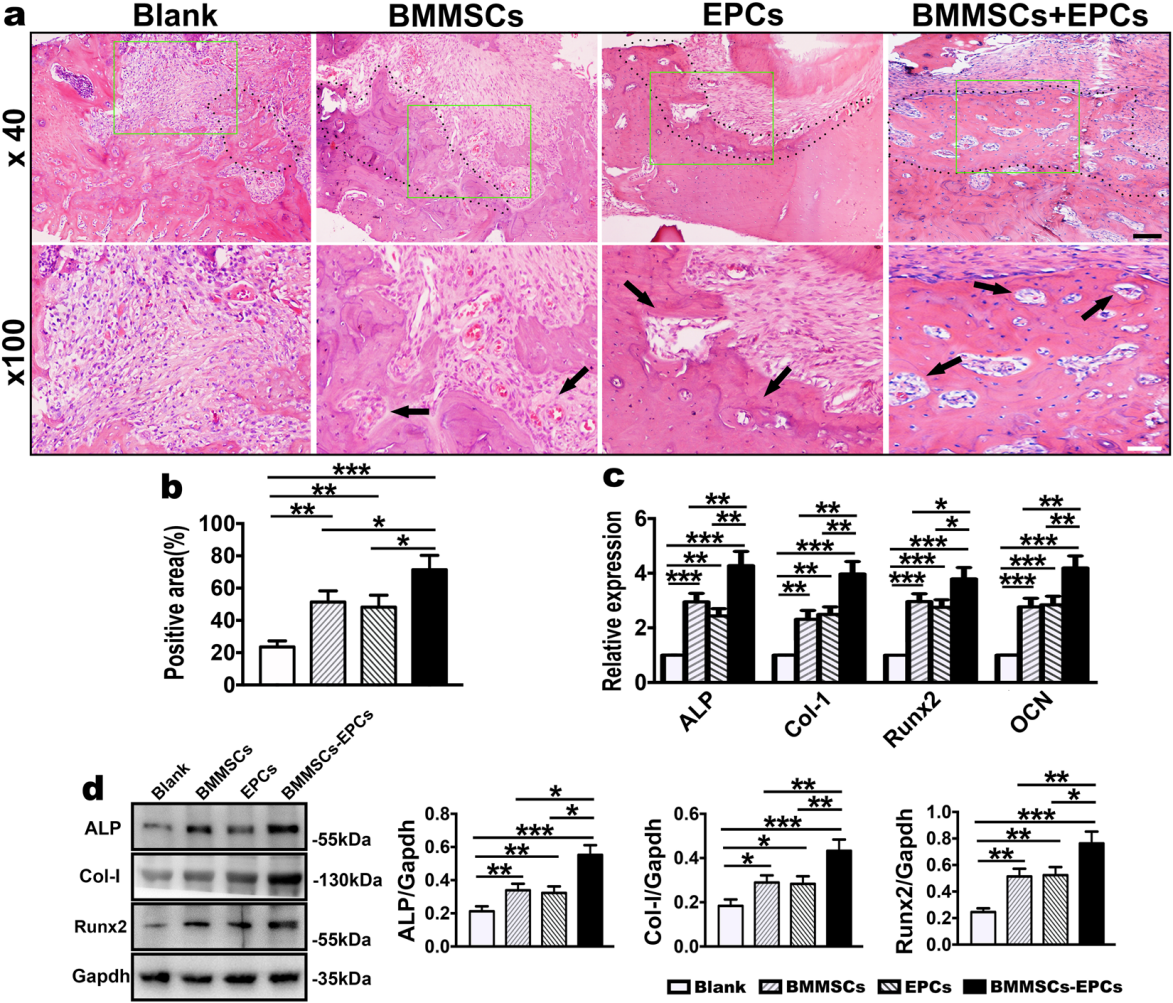
Characterization of the sheets repair effect of bone defect at 8-weeks. (**a**,**b**) H&E staining of bone defects of OVX rats treated with BMMSCs-EPCs sheet, BMMSCs sheets, EPCs sheets and control group at 8 weeks after transplantation (40× and 100×, black bar represents 200 µm and white bar represents 100 µm). The black dotted line shows newly formed bone. The black arrow shows newly formed blood vessels. (**c**) Quantitative analysis of the new bone area on H&E staining images using the Image-Pro Plus 6.0 software. (**d**) The osteogenesis-related mRNA expression of ALP, Col-1, Runx2 and OCN in BMMSCs-EPCs sheet, BMMSCs sheet and EPCs sheet at 8 weeks. (**e**) Protein expression levels of ALP, Col-1, and Runx2 by western blotting of BMMSCs-EPCs sheet, BMMSCs sheet and EPCs sheet. Data are presented as the means ± SD, n = 5. *p < 0.05, **p < 0.01 and ***p < 0.001 represent significant differences between the indicated columns.

At 2, 4 and 8 weeks, the rats were sacrificed, and the maxillae were detected by micro-CT, H&E staining, western blot and quantitative real-time PCR. Micro-CT analysis indicated that the repair of the maxillary alveolar bone defect in OVX rats with BMMSCs-EPCs sheets was better than that of BMMSCs and EPCs sheets (Fig. 4a). Further, the borders of the bone defects became indistinct and difficult to separate from the original alveolar bone. Bone histomorphometric analysis revealed that the BMMSCs-EPCs sheets group showed significant increases in BV/TV and Tb.Th (Fig. 4b,c) and reductions in Tb.Sp (Fig. 4d) compared to the blank group and the BMMSCs and EPCs sheets groups at 2, 4 and 8 weeks. Additionally, the BMMSCs sheets had an increased value of BV/TV and Tb.Th and a decreased value of Tb.Sp at all three time points. However, in these subgroups and at these time points, there was no significant difference in Tb.N (Fig. 4e). H&E staining were performed to determine whether the BMMSCs-EPCs sheet group produced new bone compared with the BMMSCs and EPCs sheet groups and the blank control group at 8 weeks (Fig. 5a). Notably, new bone trabeculae were relatively orderly, and the new bone tissue structure was similar to normal bone tissue. Moreover, there were more vascular structures in the BMMSCs-EPCs sheet group at 8 weeks (Fig. 5a). Quantitative analysis showed that the BMMSCs-EPCs sheets group formed a higher amount of new bone than did the other three groups; the same was found for the BMMSCs and EPCs sheets compared with the blank group (Fig. 5b). The mRNA expression levels of ALP, Col-1, Runx2 and OCN were significantly upregulated in the BMMSCs and EPCs sheets group compared with blank group and in the BMMSCs-EPCs sheets group compared with the other three groups at 8 weeks (Fig. 5c). Consistently, the WB assay demonstrated more upregulated protein expression of ALP, Col-1 and Runx2 in the BMMSCs-EPCs sheets group than in the BMMSCs and EPCs sheets group (Fig. 5d).

## Discussion

The findings of the present study indicated that BMMSCs-EPCs sheets demonstrated higher osteogenic and angiogenic potential in the regeneration of alveolar bone defects in OVX rats compared to the BMMSCs and EPCs sheet groups and the blank control group. Additionally, the structure of the new bone regenerated by the BMMSCs-EPCs sheet was similar to normal bone tissue. Osteoporosis models were constructed using 12-week-old female SD rats in this study, and at 12 weeks after ovariectomy operation, micro-CT analysis showed that indicators, including BV/TV, Tb.Th and Tb.N, in the experimental group were significantly lower than those in the control group, whereas the trabecular separation were upregulated. Therefore, SD female rats showed apparent osteoporosis after ovariectomy for 12 weeks.

Bone grafts and artificial bone have often been used for the regeneration of bone defects caused by trauma, cyst, benign and malignant tumors, and congenital deformities, but these methods have several limitations, such as morbidity and complications^4,5^. Additionally, the injection of single-cell suspensions contributes to the uneven distribution and weak adhesion of cells, which may ultimately induce cell death^26^. Moreover, it is impractical to transplant isolated cells for bone regeneration in large-sized defects, which would require a great quantity of cells. The rise of tissue engineering is a better choice for the regeneration of organs or tissues that are lost or damaged. Currently, biological material does not completely meet the requirements of mechanical strength, the biological environment of bone tissue, and supporting the formation of blood vessels to restore the function of bone tissue^27–29^. BMMSCs have been the focus of tissue engineering for repairing bone defects in recent years, and it has been reported that these cells had self-renewal potential and could differentiate into osteoblasts, adipocytes, chondrocytes, neurons, and myogenic cells. BMMSCs are easy to isolate and culture. In addition, these cells have the characteristics of rapid amplification *in vitro*, adequate source and stable biocompatibility. Moreover, BMMSCs derived from postmenopausal osteoporotic individuals exhibited reduced osteogenic and increased adipogenic potential^30^.

The alveolar bone defect in the postmenopausal women were difficult to treat with BMMSC because of the lack of estrogen^24^ and our present research provided a novel treatment it. The main reason for the failure of bone healing is that the problems of vascularization are not well solved among many factors. EPCs were initially found in human peripheral blood in 1997 by *Asahara et al*.^31^, who confirmed that these cells had the ability of self-renewal and differentiating into mature endothelial progenitor cell populations. There is considerable evidence to support the existence and origin of EPCs and the role of the EPCs in the formation of new blood vessels. In fact, EPCs can proliferate, migrate and differentiate into mature endothelial cells and are involved in the processes of angiogenesis, including mobilization, migration, adhesion, and differentiation. The mobilization of EPCs from bone marrow into peripheral circulation is a crucial step in angiogenesis. EPCs and cytokines are mobilized to the defective region, differentiate into mature endothelial cells, and participate in the formation of new blood vessels under ischemia^32,33^. Moreover, *Seebach et al*.^34^ demonstrated that the coculture of EPCs and MSCs had a synergistic effect on new vessel formation and seems to be a potential osteogenic composite *in vivo* application. They found that human early EPCs directly and indirectly accelerated the early vascularization of MSC-stimulated bone regeneration of a composite after xenotransplantation into a critical-sized femoral defect in athymic nude rats. *Sebastian et al*.^35^ confirmed that EPCs or smooth muscle cell-mesenchymal stem cells (SMC-MSCs) implanted alone or in combination into PBCB-scaffolds (decalcified processed bovine cancellous bone) provoke an angiogenic response that leads to increased vascular density. Additionally, these cell types improved bone formation, indicating a positive correlation between neovascularization and bone formation. In this study, we explored whether EPCs could improve the osteogenic ability of BMMSCs in OVX rats to repair alveolar defects in the maxilla.

The cell-sheet technique is a promising approach in regenerative medicine, which has intact ECM and junctions between cells that provide mechanical support and thereby maintain the integrity of the transplant. In classical tissue engineering techniques, confluent cultures are usually harvested by using trypsin. However, this approach would destroy the ECM produced by cells. The ECM is rich in growth factors and has strong osteogenic potential. Therefore, our work was based on the results from the Okano group, who utilized cell sheet technology to allow cells to be restored in their respective matrix after transplantation^36^. Traditional bone tissue engineering involves three factors, biodegradable scaffolds, stem cells, and growth factors. However, these scaffolds still face problems of biocompatibility, mechanical properties, and bioresorbability, which are not adaptable to the properties of natural bone. Thus, a cell sheet without a scaffold was utilized in our study. To date, thin-layered tissues, such as the cornea, esophagus, articular cartilage, and periodontal tissue, are fully regenerated by the cell sheet transplantation of autologous mucosal epithelial cells (for cornea and esophagus), chondrocytes (for cartilage), and periodontal ligament-derived cells (periodontal tissue)^37^. Transplanted cell sheets not only replace the injured tissue and regain the impaired function but also deliver growth factors and cytokines over a prolonged period, which enables the promotion of tissue repair. In our study, creating BMMSCs-EPCs sheets did not require a special method and could be lifted as an intact cell sheet by a scraper. The cell sheet can maintain the integrity of the extracellular matrix to the extent, the biological characteristics of seed cells, and the normal connections between cells and can improve the effect of tissue repair^38,39^. Histological results indicated that the three types of cell sheets have osteogenic potential and that the calcium deposition created by the BMMSCs-EPCs sheet is greater than that of the BMMSCs and EPCs sheet groups, indicating that the osteogenic ability of the BMMSCs-EPCs sheet is greater than that of the BMMSCs and EPCs sheets. The *in vitro* results showed that osteogenesis-related genes, ALP, Col-1, Runx2 and OCN, in the BMMSCs-EPCs sheet were markedly higher than in the BMMSCs and EPCs sheets. Moreover, western blotting showed that the protein expression levels of ALP, Col-1, and Runx2 in the BMMSCs-EPCs sheet were significantly higher than those in the BMMSCs and EPCs sheets.

Because micro-CT is a useful and reliable method for evaluating bone healing, it was used for radiological analysis in the current study. According to the findings of micro-CT analysis, new bone formation in the maxillary alveolar bone defects increased over time and enhanced bone regeneration in the BMMSCs-EPCs sheet group compared with that in the BMMSCs sheet group, EPCs sheet group and control group. Moreover, the highest values for the BV/TV and Tb.Th ratio measured by micro-CT quantitative analysis were observed in the BMMSCs-EPCs sheet group. However, the EPCs sheet have comparable capacity with BMMSCs sheet in repairing of the alveolar defects. Studies found that EPCs are mobilized from the bone marrow into circulation and migrate to affected areas to aid in tissue recovery^40^. Traditionally, endothelial and osteoblastic cells have been thought to be derived from two separate progenitor lines; however, increasing evidence suggests that there may be an overlap between these two cell lines. This potential role of EPCs in bone formation is further supported by research that shows these cells can be differentiated into osteoblastic cells^41,42^. Meanwhile, we found that the osteogenic markers ALP, Col-1, Runx2 were highly expressed in the BMMSCs-EPCs sheet group. Histological analysis of new bone showed the new bone formation in BMMSCs-EPCs sheet group was higher than that in the other three groups, indicating better a repair effect in the BMMSCs-EPCs sheet group. The enhanced bone formation might be due to the delivery of osteogenic cells and ECM to defect sites by BMMSCs-EPCs sheets.

In summary, early vascularization in alveolar bone defects is a prerequisite for bone regeneration in the osteoporosis rat. BMMSCs and EPCs can be transferred as a sheet without exogenous scaffolds into the alveolar bone defect of OVX rats using a simple and effective cell sheet technique, and the data showed that the combination of both cell types strongly enhanced the healing of defects compared to the other alternatives, likely due to the higher osteogenic potential of BMMSCs-EPCs sheets. We demonstrate that EPCs may dramatically improve the vascularization of MSC-driven bone regeneration. Altogether, BMMSCs-EPCs sheets without exogenous scaffolds represent a promising strategy to repair alveolar bone defects in OVX rats and may lay the foundation for clinical application in the future. However, additional studies are needed to investigate the mechanism of the improvement of the repair of alveolar bone defects by BMMSCs-EPCs sheets and to examine the effects on larger bone defects.

## Materials and Methods

### Experimental animals, ethics statement and ovariectomized models

Experiments were performed on seventy-eight female Sprague Dawley (SD) rats aged 12 weeks and weighting 200~220 g provided by the animal center of the Fourth Military Medical University in Xi’an, China. The animal welfare and all procedures were performed according to the Guide for the Care and Use of Laboratory Animals (Ministry of Science and Technology of China, 2006) and approved by the Ethics Committee of the Fourth Military Medical University. For ovariectomized models, twelve 12-week-old female SD rats were randomly divided into experimental and control groups (n = 6). In the experimental group, six rats were intravenously injected with 1% sodium pentobarbital at 0.35 ml/100 g for anesthetic premedication. In a sterile environment, the rats were treated with classical methods to remove bilateral ovaries. In the control group, six rats were operated to remove a small amount of fat as a control. Intraperitoneal injection of antibiotics once a day to prevent infection was administered for three days after the operation. Three months later, we removed the bilateral tibias of two groups and shaved soft tissue under aseptic environment after the animals were sacrificed under anesthesia, and subsequently we employed Micro-CT scanning to compare the two groups indicators containing trabecular thickness (Tb.Th), trabecular number (Tb.N), trabecular spacing (Th.Sp) and bone volume fraction (BV/TV).

### Isolation and culture of BMMSCs and EPCs

Six 12-week-old female SD rats were sacrificed and subsequently soaked in 75% alcohol for 30 min to isolate the primary cells. The tibias and femora of the rats were collected for the isolation of BMMSCs and EPCs. The bone marrow cavities were repeatedly flushed with Minimum Essential Medium alpha Medium (α-MEM) (Gibco, Rockville, MD, USA), containing 100 U/ml penicillin and 100 mg/ml streptomycin (Sigma-Aldrich, Boston, MA, USA), into 25-cm^2^ flasks using a syringe. Then, the bone marrow was transferred into a centrifuge tube containing 7 ml of Percoll solution (1.073 g/ml) (GE Healthcare, USA), and isolated by density gradient centrifugation at 2000 rpm for 20 min. The middle layer (mononuclear cells) was extracted and subsequently transferred into a centrifuge tube followed by centrifugation at 1000 rpm for 10 min. The supernatant was removed, and the bone marrow was incubated at 37 °C in 95% humidified air containing 5% CO_2_ in α-MEM medium supplemented with 10% fetal bovine serum (FBS, Gibco, USA). Forty-eight hours later, the adherent cells were BMMSCs.

The mononuclear cells were washed twice with PBS, and then suspended in Endothelial Cell Basal Medium-2 supplemented with EGM-2 SingleQuot Kit Suppl. & Growth Factors (Lonza Walkersville, MD, USA). After seeded at 37 °C in 95% humidified air containing 5% CO_2_ for 48 h, non-adherent cells were collected and re-suspended in EGM-2 medium. To investigate the endothelial progenitor characteristics of the EPCs, the binding of FITC-UEA-1 and uptake of DiI-Ac-LDL by the cells were analyzed by dual staining of the cells. EPCs were washed twice with PBS and incubated with 2.5 mg/L DiI-Ac-LDL for 1 h in the dark. Thereafter, the cells were washed twice with PBS and fixed with 2% paraformaldehyde. Then, the cells were incubated with 10 mg/L FITC-UEA-1 in the dark. After final washing with PBS, the cells were analyzed for the uptake of Dil-Ac-LDL and binding of FITC-UEA-1 by inverted fluorescence microscopy.

### Flow cytometry

BMMSCs from passage two were suspended in 400 μl of PBS and incubated with each specific antibody. To evaluate surface markers, phycoerythrin (PE)-coupled antibodies against CD29 (25-0291-80, eBioscience, USA), rabbit anti-CD34 (ab81289, Abcam, Cambridge, UK), rabbit anti-CD45 (ab10558, Abcam, Cambridge, UK), mouse anti-CD90 (ab225, Abcam, Cambridge, UK) and mouse anti-CD105 (ab11411, Abcam, Cambridge, UK) were used. The secondary antibodies used in the present study included Cy3-conjugated goat anti-rabbit IgG and goat anti-mouse IgG (Zhuangzhi Co. Ltd, Xi’an, China). After incubation in the dark, the cells were washed with PBS and then resuspended in 400 μl of PBS. Cell fluorescence was determined using a flow cytometer (BD Biosciences, San Jose, CA, USA).

Primary EPCs were suspended in 400 μl of PBS and incubated with each specific antibody. Rabbit anti-CD34 (ab81289, Abcam, Cambridge, UK), rabbit anti-CD133 (ab19898, Abcam, Cambridge, UK) and rabbit anti-VEGFR2 (ab2349, Abcam, Cambridge, UK) were used to identify surface markers. The secondary antibodies used in the present study included Cy3-conjugated goat anti-rabbit IgG (Zhuangzhi Co. Ltd, Xi’an, China). After incubation at 4 °C in the dark, the cells were washed with PBS and then resuspended in 400 μl of PBS. Cell fluorescence was determined using a flow cytometer (BD Biosciences, San Jose, CA, USA).

### Detection of multi-lineage differentiation ability of BMMSCs

BMMSCs at passage two were inoculated into 6-well plates. For osteogenesis studies, after 24 hours, the cells were adhered and the medium was switched to osteoinductive differentiation medium (α-MEM culture medium containing 10% FBS, β-glycerophosphate 10 mM, ascorbic acid 50 mg/L, dexamethasone 10 nM). The media was changed every two days. After osteogenic induction for 21 days, we detected the formation of mineralized nodule by using alizarin red staining. For adipogenesis studies, BMMSCs at passage three were inoculated onto 6-well plates. After cells adhesion, adipogenic induction medium (α-MEM containing 10% FBS, indomethacin 200 μM, IBMX 0.5 mM, insulin 10 μM and dexamethasone 1 μM) was used in place of the original culture medium. The medium was changed every two days. After 14 days, the lipid droplets were stained by Oil Red O solution.

### Fabrication and histological observations of cell sheets

**Figure 6:**
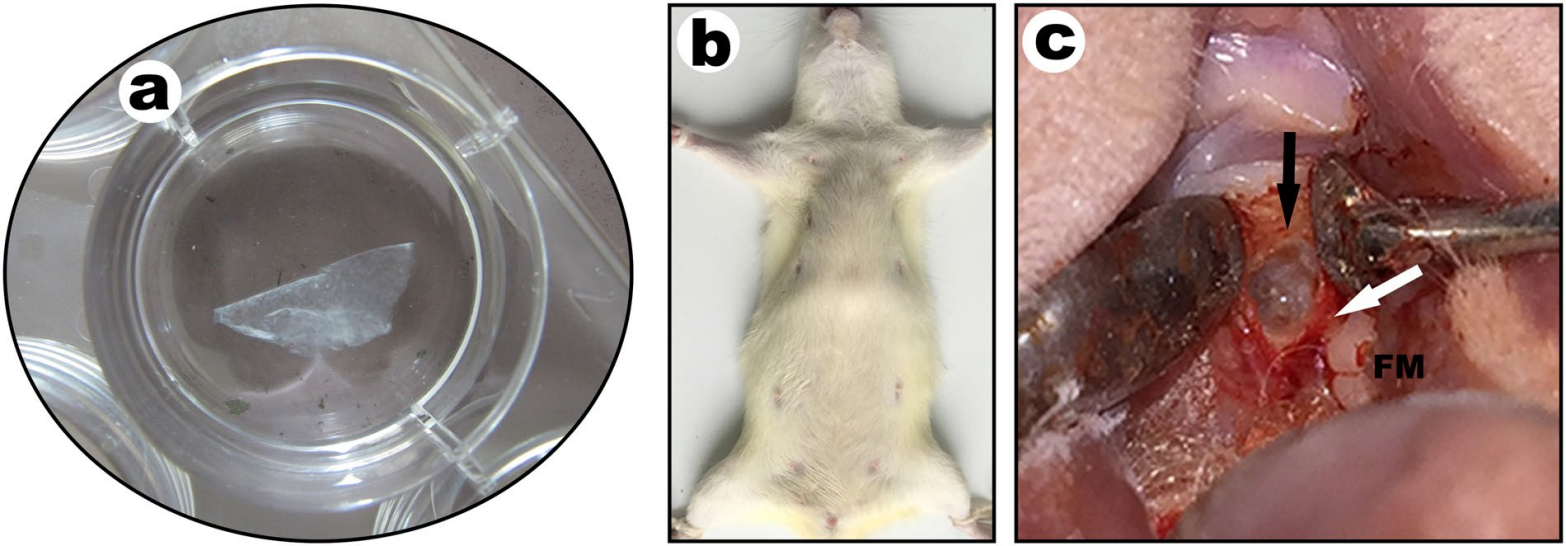
The harvested cell sheet and bone defect in OVX rat. (**a**) Harvesting the cell sheet. After 2 weeks of culturing in induction medium, a cell sheet formed and could be lifted intact from the dish by using forceps. (**b**) The rat position of alveolar bone defect model. (**c**) The bone defect was prepared in the mesial-lingual side of the maxillary first molar mesiolingual root in each OVX rat. Black arrow, the defect; White arrow, mesiolingual root; FM, first molar.

BMMSCs-EPCs sheets were created by culturing BMMSCs with an equal number of EPCs were seeded at a density of 1 × 10^5^ cells/cm^2^ onto six-well plates containing α-MEM culture medium with 10% fetal bovine serum at 37 °C in an atmosphere of 5% CO_2_ and 95% air for the first 24 hours. Subsequently, α-MEM culture medium was substituted for the medium, which included 10% FBS and ascorbic acid 50 mg/L in α-MEM culture medium. Ten days later, the BMMSCs-EPCs sheet was prepared, and the cell sheet was lifted by a scraper (Fig. 6a). The methods for forming BMMSCs and EPCs sheets were same as those used for the BMMSCs-EPCs sheet. Finally, the cell sheets were fixed in 4% paraformaldehyde, paraffin-embedded and sliced at 4 μm for histological staining.

### Alveolar bone defect and cell sheets transplantation

Sixty 12-week-old female SD rats were ovariectomized as previously described and randomly assigned into four groups according to the type of implant received (n = 15 in each group). The blank group was a control, BMMSCs group and EPCs group received the BMMSCs and EPCs sheet respectively and BMMSCs-EPCs group received the BMMSCs-EPCs sheet. OVX rats were intravenously injected with 1% sodium pentobarbital at 0.35 ml/100 g for anesthetic premedication. An alveolar bone defect was prepared with a 2.3-mm diameter round carbide bur (8#CARBIDE BURS, Mani, Japan) in the mesial-lingual side of the left maxillary first molar mesiolingual root in each osteoporotic rat and ensured the size of each defect was same as the round bur (Fig. 6b,c). The maxillary alveolar bone defects should be created in a slow and intermittent way in the process of drilling to prevent alveolar bone necrosis caused by excessive heat produced during the preparation of the defects. The BMMSCs-EPCs, BMMSCs and EPCs sheets were lifted up by a scraper and then implanted into the defects in maxillary alveolar bone. After implantation, the mucosa was carefully closed to avoid leakage of the cell sheets. Group D underwent surgery without implantation as a control. Animals were sacrificed at 2, 4 and 8 weeks after surgery, respectively, in the three experimental groups and the control group (n = 5), and the maxillae were prepared for micro-CT analysis and histological staining to evaluate the effect of the repair of alveolar bone defects.

### Histological staining and alkaline phosphatase (ALP) activity assay

The cell sheets were fixed in 10% formalin and embedded in paraffin. 4 μm sections were prepared consecutively for H&E staining and ALP staining (PMC-AK20-COS, Cosmo Bio Co., USA) following the manufacturer’s protocol. For the ALP activity assay, cell sheets were lysed by RIPA lysis buffer (Beyotime, China), and the cell supernatant was collected into a 96-well plate. The substrates and p-nitrophenol from the Alkaline Phosphatase Assay Kit (Beyotime, China) were added and the ALP activity was detected at the wavelength of 405 nm.

### RNA Extraction, Reverse Transcriptase PCR and Quantitative Real-time PCR

Quantitative real-time PCR was used to detect the gene expression of BMMSCs induced to osteoblasts and adipocytes. Total RNA was extracted using Tripure Reagent (Roche, Basel, Swiss) according to the manufacturer’s instructions, followed by reverse-transcriptase (RT)-PCR and cDNA synthesis. The analysis was performed with 1 µg of total RNA, using oligo-deoxythymidine primers (Roche Diagnostics, Basel, Swiss) in a 20-µl volume at 37 °C for 20 min. The gene expression was analyzed by quantitative real-time PCR (qRT-PCR) using SYBR® Premix Ex Taq^TM^ II (RR820A, Takara, Japan) in the CFX96 Real-time PCR machine (Bio-rad, Hercules, CA, USA). The qRT-PCR program was set at 94 °C for 5 min; 35 cycles of 94 °C for 45 s, 57 °C for 45 s, and 72 °C for 1 min; followed by 72  °C for 1 min. The qRT-PCR was conducted with GAPDH as the house-keeping gene, and the mean values were derived using the formula 2^−ΔΔCt^. The primer sequences are detailed in Table 1.

**Table 1:** Gene primers.

Genes	Forward Primer (5′–3′)	Reverse Primer (3′–5′)
*BSP*	AATGAAAACGAAGAAAGCGAAG	ATCATAGCCATCGTAGCCTTGT
*OCN*	AAGGTGGTGAATAGACTCCG	AAACGGTGGTGCCATAGATG
*ALP*	CACGTTGACTGTGGTTACTGCTGA	CCTTGTAACCAGGCCCGTTG
*LPL*	TCTTCAACTGGCTGGAGGAAG	TATGCCTTGCTGGGGTTTTCT
*PPAR-γ*	CATTTCTGCTCCACACTATGAA	CGGGAAGGACTTTATGTATGAG
*Col-1*	ATCACCAGACGCAGAAGTCATA	ACCAGGAGGACCAGGAAGTC
*Runx2*	CCTCCTGCTTCTCCCTTTATG	AACACACAGCCAACTCAAACAC
*GAPDH*	GGCACAGTCAAGGCTGAGAATG	ATGGTGGTGAAGACGCCAGTA

### Western blotting analysis

Western blotting was used to detect the differences in protein expression between three types of cell sheets: BMMSCs-EPCs, BMMSCs, and EPCs. Protein extraction was performed using radio immunoprecipitation assay (RIPA) lysis buffer containing protease inhibitor cocktail (Sigma-Aldrich, Boston, MA, USA). The protein concentrations were determined using the bicinchoninic acid (BCA) assay (Thermo Scientific, Waltham, MA). The proteins were then separated by 10% sodium dodecyl sulfate-polyacrylamide gel electrophoresis (SDS-PAGE; Bio-Rad, Hercules, CA). The separated proteins were transferred onto a polyvinylidene fluoride (PVDF) membrane (Bio-Rad). The primary antibodies included rabbit anti-ALP antibody (ab108337, Abcam, Cambridge, UK), mouse anti-Col-1 (ab90395, Abcam, Cambridge, UK), goat anti-Runx2 (sc8566, Santa Cruz, CA, USA). After several washes in Tris-buffered saline (Bio-Rad) with 0.2% Tween (TBST), the membranes were incubated with the corresponding HRP-conjugated goat anti-rabbit IgG, goat anti-mouse and donkey anti-goat secondary antibodies (Zhongshan Inc., Beijing, China) and then developed using electrochemiluminescence (ECL) agents (Millipore, Darmstadt, Germany). To compare the relative protein intensity of each group, loading was normalized using GAPDH (ab181602, Abcam, Cambridge, UK) expression before analysis. The gray value of the protein was measured by the ImageJ software.

### Statistical analysis

Statistical analysis was conducted by SPSS 22.0 software (SPSS Inc., Chicago, IL, USA). The acquisition and analysis of all data were completed blind. As per normal procedures, the data distribution was tested with the Shapiro-Wilk test at a 95% confidence level, and Levene’s test was used to assess homogeneity of variance. Student’s t-test was used to compare two groups. For multiple comparisons of three or more groups, one-way analysis of variance (one-way ANOVA) with Tukey’s post hoc test was used. The results are expressed as the means ± standard deviation for each group. P values less than 0.05 were considered statistically significant in all cases. All experiments were repeated at least three times.

## Electronic supplementary material

Supplementary Figure
