# Supplementary material for: Evaluation of BMMSCs-EPCs sheets for repairing alveolar bone defects in ovariectomized rats
Source: Sci Rep. 2017 Nov 29;7:16568. doi: 10.1038/s41598-017-16404-3 (PMC5707386; doi:10.1038/s41598-017-16404-3)
Supplement: Supplementary file 1 — Supplementary Figure [file 41598_2017_16404_MOESM1_ESM.pdf]

Evaluation of BMMSCs-EPCs sheets for repairing alveolar bone defects in ovariectomized rats

Yi Wen[1, a], Hongxu Yang[2, a], Yanli Liu[3, a], Qian Liu[1], Axian Wang[1], Yin Ding[1], Zuolin Jin[1]*

1. State Key Laboratory of Military Stomatology and National Clinical Research Center for Oral Diseases and Shaanxi Clinical Research Center for Oral Diseases, Department of Orthodontics, School of Stomatology, the Fourth Military Medical University, 145 Changle West Road, Xi'an 710032, China.
2. State Key Laboratory of Military Stomatology and National Clinical Research Center for Oral Diseases and Shaanxi International Joint Research Center for Oral Diseases, Department of Oral Anatomy and Physiology and TMD, School of Stomatology, the Fourth Military Medical University, 145 Changle West Road, Xi'an 710032, China.
3. State Key Laboratory of Military Stomatology and National Clinical Research Center for Oral Diseases and Shaanxi International Joint Research Center for Oral Diseases, Department of General Dentistry and Emergency, School of Stomatology, the Fourth Military Medical University, 145 Changle West Road, Xi'an 710032, China.

*Corresponding author: zuolinj@fmmu.edu.cn

[a]These authors contributed equally to this work.

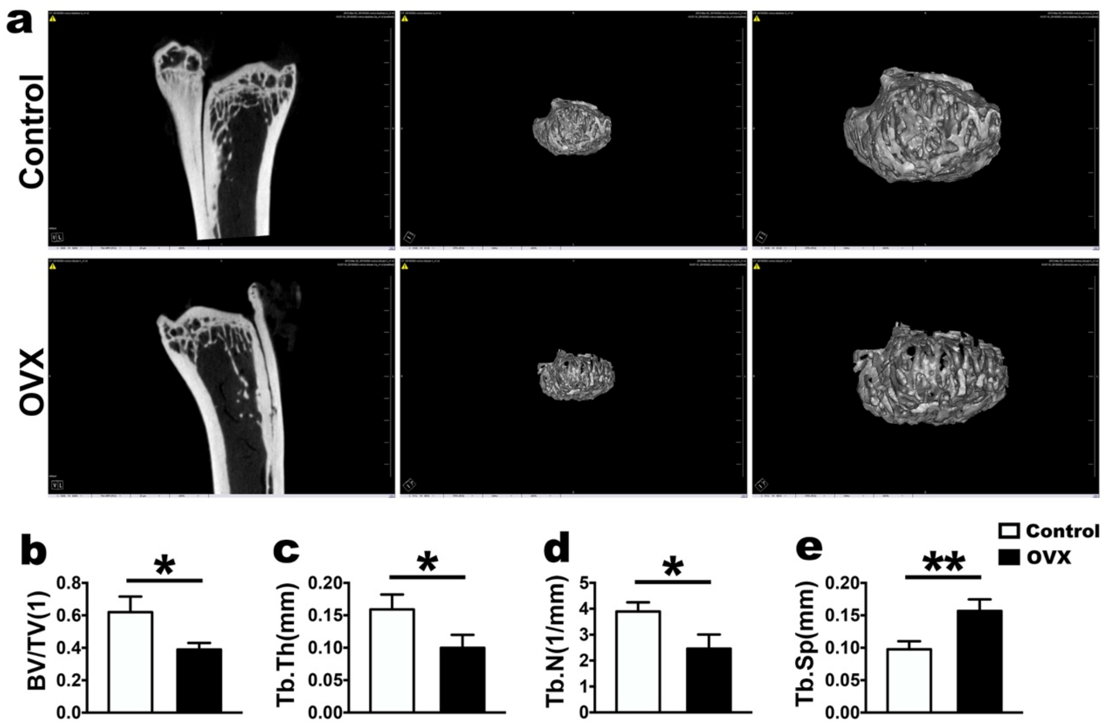

Supplementary Figure 1. Examination of osteoporosis model. (a) Schematic of the areas scanned by micro-CT. The proximal tibial metaphysis was scanned starting at approximately 1 mm distal to the growth plate. (b) BV/TV, (c) Tb.Th and (d) Tb.N in OVX group were lower than that in control group. (e) Tb.Sp in control group was lower than that in the experimental group. Data are presented as the means ±SD, n = 6. * $p < 0.05$ and ** $p < 0.01$ represent significant differences between the indicated columns.

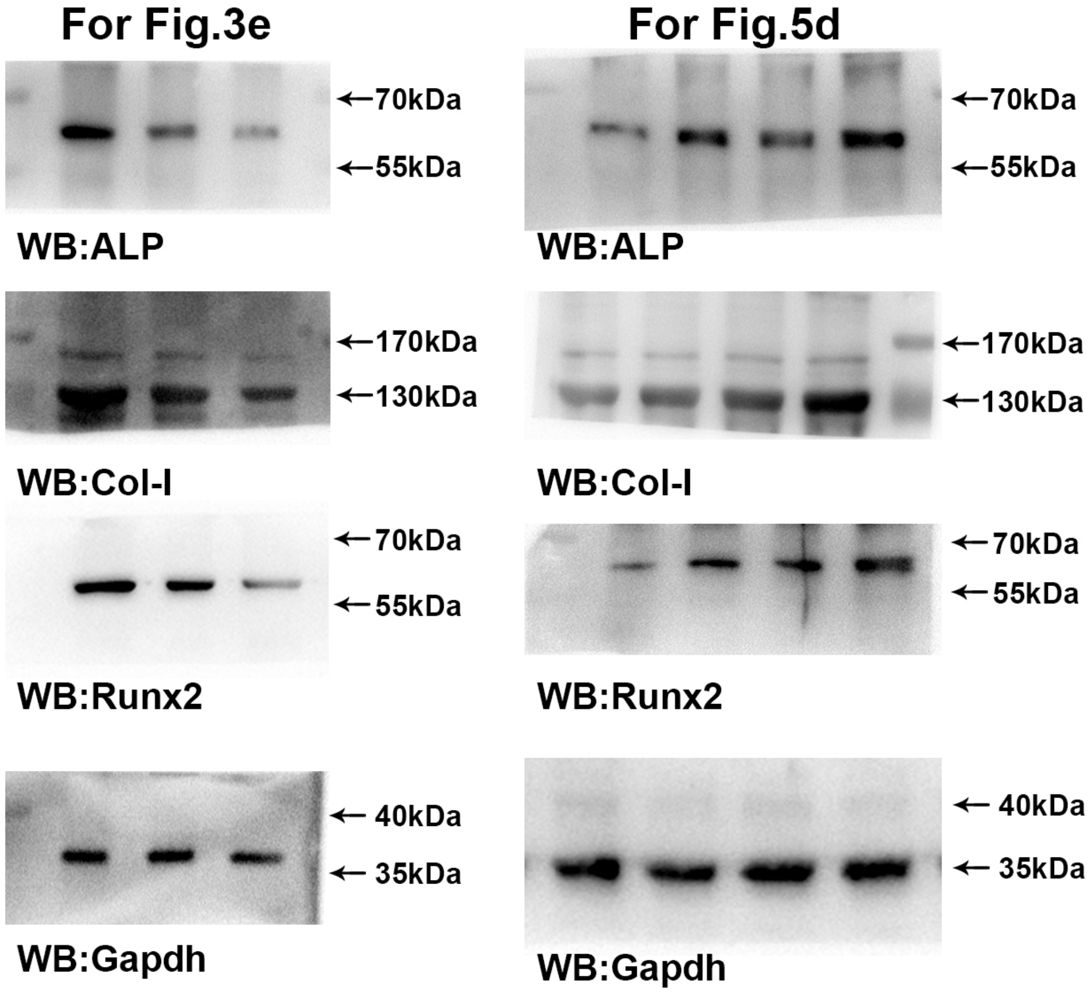

Supplementary Figure 2. Uncropped picture of Western blots results in Figure 3e and Figure 5d. Arrows indicate the size marker.
